# SPP1 macrophages across diseases: A call for reclassification?

**DOI:** 10.1096/fj.202403227R

**Published:** 2025-03-06

**Authors:** Alessio Reggio, Claudia Fuoco, Rebecca Deodati, Alessandro Palma

**Affiliations:** ^1^ Saint Camillus International University of Health Sciences Rome Italy; ^2^ Department of Biology University of Rome Tor Vergata Rome Italy; ^3^ Department of Biology and Biotechnologies “Charles Darwin” Sapienza University of Rome Rome Italy

**Keywords:** cancer, inflammation, macrophages, SPP1

## Abstract

SPP1+ macrophages, characterized by elevated expression of the osteopontin gene (secreted phosphoprotein 1, SPP1), have emerged as key players in various pathological contexts, including aging, chronic inflammatory diseases, and cancer. While frequently classified as a subclass of tumor‐associated macrophages in oncological settings, their presence in noncancer conditions, such as aging‐related disorders and muscular diseases, suggests a broader role beyond tumors. These macrophages share conserved traits, including fibrosis promotion, extracellular matrix remodeling, and immune modulation, often linked to poor clinical outcomes. This perspective explores the multifaceted roles of SPP1+ macrophages across diseases and advocates for their reclassification as a distinct macrophage subtype associated with chronic or prolonged inflammation. Recognizing their cross‐disease relevance could reshape macrophage biology and inform targeted therapeutic strategies.

**Abbreviations**
ARG1: arginase 1
CAF: cancer‐associated fibroblast
CCL15: C‐C motif chemokine ligand 15
CCR2: C‐C motif chemokine receptor 2
CD163: cluster of differentiation 163
CD206: cluster of differentiation 206
CD44: cluster of differentiation 44
CD80: cluster of differentiation 80
CD86: cluster of differentiation 86
CD9: cluster of differentiation 9
ECM: extracellular matrix
FAP: fibro‐adipogenic progenitor
IL1B: interleukin 1 beta
IL1R: interleukin 1 receptor
LGALS3: galectin 3
LGMN: legumain
LMAM: liver metastasis‐associated macrophages
MERTK: MER proto‐oncogene, tyrosine kinase
PDGFRα: platelet‐derived growth factor receptor alpha
SAMac: scar‐associated macrophages
SPP1: secreted phosphoprotein 1
TAM: tumor‐associated macrophage
TGFβ: tumor growth factor beta
TLR4: toll‐like receptor 4
TNF: tumor necrosis factor
TNFSF12: tumor necrosis factor superfamily member 12
TREM2: triggering receptor expressed on myeloid cells 2

## 1 INTRODUCTION

**FIGURE 1:**
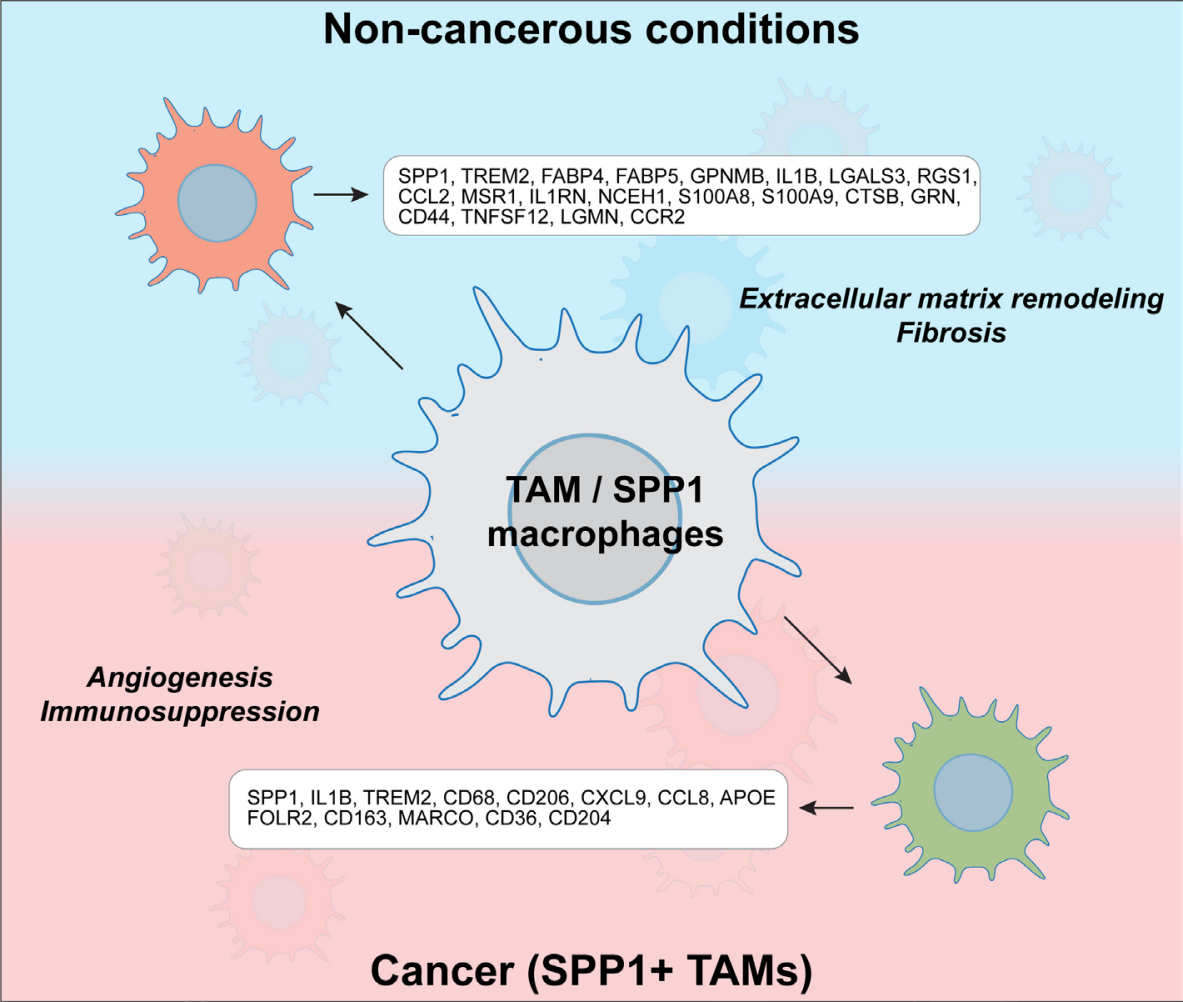
Macrophages expressing high levels of secreted phosphoprotein 1 (SPP1) are considered a subclass of tumor‐associated macrophages (TAMs). They also play fundamental roles in other noncancerous conditions, expressing a wide range of gene markers. These markers have been mostly identified through bulk and single‐cell RNA sequencing experiments. The picture illustrates some of the biomarkers characterizing SPP1+ macrophages in different conditions (cancer and noncancer diseases).

Recent advances in macrophage biology have revealed a remarkable diversity among these immune cells, highlighting the existence of specialized subpopulations with distinct functional roles in health and disease. Among these, SPP1+ macrophages, characterized by elevated osteopontin (SPP1) expression, have garnered significant attention due to their consistent association with pathological states. Originally identified in cancer as tumor‐associated macrophages (TAMs), SPP1+ macrophages have since been implicated in various conditions, including aging, chronic inflammatory disorders, neurodegenerative diseases, and tissue remodeling (Figure 1).

Their conserved traits, such as promoting fibrosis, remodeling the extracellular matrix, and modulating immune responses, suggest they play a pivotal role in sustaining chronic inflammation and tissue dysfunction. Furthermore, their presence often correlates with poor clinical outcomes, underscoring their relevance as potential therapeutic targets. Despite these shared characteristics, SPP1+ macrophages exhibit functional adaptability across different disease contexts, raising questions about their classification and the underlying mechanisms that drive their diverse roles.

In this perspective, we briefly summarize recent discoveries on the multifaceted roles of SPP1+ macrophages across various pathological conditions, emphasizing their shared traits and the critical differences dictated by the tissue microenvironment and pathological inflammatory context. Based on our comparative literature investigation, we also propose a re‐evaluation of their classification, advocating for their recognition as a distinct macrophage subtype linked to prolonged inflammatory states rather than specific to tumors. Such a shift in perspective could not only advance our understanding of macrophage biology but also open new avenues for targeted therapeutic interventions.

### 1.1 Aging and noncancer diseases: The rise of SPP1+ macrophages

Aging presents a compelling context in which SPP1+ macrophages emerge as key players. Single‐cell RNA sequencing studies have revealed their abundance in the skeletal muscle of aged mice, where they exhibit hallmarks of senescence and enhanced angiogenic and lipid metabolic activity.^1^ These findings are echoed in other studies linking SPP1+ macrophages to chronic inflammation and tissue degeneration in aging muscle.^2^ Strikingly, a similar macrophage population is implicated in the severe phenotype of Duchenne Muscular Dystrophy, underscoring their role in maintaining pro‐fibrotic and inflammatory microenvironments.^3^, ^4^, ^5^ Beyond musculoskeletal systems, SPP1+ macrophages also influence neurodegenerative diseases. In Alzheimer's disease, an upregulation of SPP1‐positive microglia correlates with inflammation and synaptic loss. Perivascular macrophages with SPP1 profiles modulate microglial phagocytic activity, offering a potential mechanism underlying synapse degradation.^6^, ^7^ This dual contribution to inflammation and neurodegeneration positions SPP1+ macrophages as central figures in aging‐related pathologies.

Intriguingly, similar profiles have been observed in rheumatoid arthritis, where SPP1 production in response to extracellular calcium drives inflammation.^8^ In white adipose tissue remodeling, SPP1+ macrophages recruit PDGFRα+ progenitor cells via CD44 signaling, highlighting their chemoattractive and immunomodulatory capacities.^9^ Such recruiting and modulatory properties toward mesenchymal progenitors largely contribute to the establishment of fibrosis throughout the coronary perivascular adipose tissue, aggravating the progression of atherosclerosis.^10^

Further noncancerous diseases in which this macrophage population has been identified include myocardial infarction. Here, platelet‐derived signals stimulate the differentiation of SPP1+ macrophages. These macrophages subsequently expand and contribute to post‐infarction fibrosis by promoting extracellular matrix remodeling and collagen deposition.^11^ Similarly, in liver cirrhosis, a distinct population of CD9‐positive, TREM2‐positive macrophages, termed scar‐associated macrophages (SAMacs), also exhibits high levels of SPP1 expression. These macrophages have been shown to expand and act as key pro‐fibrogenic mediators, fostering the progression of hepatic fibrosis.^12^

In noncancerous diseases, SPP1+ macrophages exhibit significant heterogeneity within their subclass (Figure 1). For example, in idiopathic pulmonary fibrosis, these macrophages are characterized by the expression of *MERTK*.^13^ In contrast, during COVID‐19‐induced acute respiratory distress syndrome, SPP1+ macrophages co‐express *CD163* and *LGMN* alongside *SPP1*.^14^ Similarly, in liver cirrhosis, SPP1+ macrophages express markers such as *TREM2, IL1B, LGALS3, CCR2*, and *TNFSF12*,^12^ some of which—like *LGALS3* and *TREM2*—are also observed in murine SPP1+ macrophages associated with dystrophic conditions.^4^

In all these reports referring to SSP1+ macrophages, fibrosis emerges as a unifying feature across various noncancer disease contexts. Such findings underscore that the pathological role of SPP1+ macrophages extends beyond isolated disease paradigms. In noncancer diseases, fibrosis and immune suppression appear to be recurring hallmarks of this macrophage population, suggesting that these cells play a pivotal role in orchestrating tissue remodeling and immune modulation across diverse pathological contexts. Exploring the underlying mechanisms of these shared features could unveil therapeutic targets to mitigate fibrosis and restore immune homeostasis in a wide array of chronic and acute diseases.

### 1.2 Cancer: The paradigm of tumor‐associated macrophages

The link between SPP1+ macrophages and cancer is well established, with TAMs representing the archetype of this subpopulation. TAMs are instrumental in shaping immunosuppressive tumor microenvironments, promoting angiogenesis, and facilitating extracellular matrix (ECM) remodeling. In liver cancer, for instance, resident Kupffer cells can transition into liver metastasis‐associated macrophages (LMAMs) with high SPP1 expression, driving cancer stemness through vitronectin and CCL15 signaling pathways.^15^, ^16^ Similar macrophage populations have been identified in pancreatic cancer, head and neck carcinomas, and colorectal cancer, where they foster inflammation, fibrosis, and tumor progression.^17^, ^18^, ^19^, ^20^ The IL1R pathway has also been linked to pancreatic cancer, where it characterizes a TAM population opposite to the SPP1 macrophages, playing a major role in the inflammatory response.^21^

In breast cancer, the interplay between SPP1+ macrophages and CD44‐expressing malignant cells underscores their influence on chemoresistance and recurrence.^22^, ^23^ The functional overlap of SPP1+ macrophages across cancer types highlights their potential as universal mediators of tumor progression and therapeutic resistance.

It remains to be established which molecular and cellular features distinguish SPP1+ TAMs as a unique subpopulation compared to other TAM subtypes. While many single‐cell studies have identified this macrophage subpopulation, others have not, instead reporting macrophage populations that lack SPP1 expression but share similar gene signatures and functional roles. Broadly, SPP1+ TAMs appear to be more closely linked to angiogenesis, whereas SPP1‐negative TAMs are predominantly associated with phagocytosis.^24^ The ability of SPP1+ TAMs to interact with fibroblasts and vasculature suggests a strong role in extracellular matrix remodeling and promoting angiogenesis in tumor settings.^25^ However, it is crucial to acknowledge that transcriptomics alone may not fully capture the plasticity of macrophages, as technical limitations can hinder functional features. Further studies focusing on functional characterization are needed to elucidate the defining features that make this macrophage subpopulation distinct.

### 1.3 Shared and distinct traits, and implications across pathologies

SPP1+ macrophages, whether in aging, chronically inflamed tissues, or tumors, share a core repertoire of functions while retaining distinct characteristics.

The role of osteopontin secreted by macrophages remains incompletely understood and warrants further investigation. Notably, it exists in two distinct post‐translational isoforms: one secreted into the extracellular space and the other retained intracellularly. Additionally, its ability to bind to its cognate receptor, CD44, is highly dependent on the specific isoform of the receptor expressed by the target cell. This receptor specificity is influenced by the retention of particular binding regions, which arise from alternative splicing during the transcription of the *CD44* gene.^26^ Osteopontin is known to regulate processes like immune response, cell adhesion, and migration, and tumorigenesis in cancer settings.^27^ In particular, its intracellular isoform participates in cytoskeletal rearrangement and signaling pathways initiated in response to the activation of immune receptors.^28^ Furthermore, osteopontin has been reported to activate latent TGFβ peptide in fibroblasts of dystrophic models.^29^

However, while SPP1 is a prominent marker of these macrophages, it is not the sole gene or protein defining their identity and function. Indeed, macrophages operate in complex, heterogeneous microenvironments where they both secrete and respond to diverse molecular cues. It is likely that the role of SPP1 and the role of this macrophage population are influenced by a combination of signals rather than acting as a singular defining factor for this macrophage population.

The elevated number of SPP1+ macrophages that are observed in the course of certain disease conditions enables these cells to mediate fibrosis, remodel the ECM, and suppress immune responses. This macrophage population frequently exhibits M2‐like polarization, expressing immunosuppressive markers such as CD206 and ARG1.^11^, ^16^ Such characteristics may not only facilitate chronic inflammation but also impair the body's ability to activate effective anti‐tumor or anti‐inflammatory immune responses.

At the same time, SPP1+ macrophages express pro‐inflammatory genes, including TNF,^11^ IL‐1β,^30^ and M1‐like surface markers (CD80, CD86, and TLR4).^31^ This profile characterizes a macrophage population seemingly locked in a hybrid polarization state.

Of particular interest is their interaction with fibroblasts, which seems to be a shared trait in many different disease contexts. In tumors, TAMs engage cancer‐associated fibroblasts (CAFs) to remodel the ECM,^32^ while in skeletal muscle, SPP1+ macrophages collaborate with fibro‐adipogenic progenitors (FAPs),^4^, ^5^ which are known to determine fibrosis in dystrophic contexts.^33^, ^34^ Their involvement in matrix remodeling demonstrates a pivotal pathological function for this macrophage population, thereby offering the potential for therapeutic intervention through targeting these interactions.

While numerous single‐cell RNA sequencing studies have identified shared biomarkers for these cells, they have also revealed the expression of distinct, context‐specific genes. Such variability may partly stem from technical factors, including sequencing depth or clustering resolution. However, it may also reflect the intrinsic functional diversity of these macrophages across different pathological settings, shaped by factors such as the disease environment, stage of progression, and interactions within the cellular microenvironment.

Their paradoxical Janus‐like behavior, promoting fibrosis and immune suppression on one hand while being linked to chronic inflammation on the other, suggests a more integrated role for SPP1+ macrophages in coordinating immune responses and tissue remodeling. This raises the intriguing possibility that these macrophages act as a nexus connecting fibrosis, immune suppression, and chronic inflammation. However, unraveling this potential link will require further detailed experimental investigations.

### 1.4 Rethinking macrophage classification

The discovery of SPP1+ macrophages in both cancer and noncancer conditions raises fundamental questions about the current classification of macrophages. Although SPP1+ macrophages are frequently designated as TAMs (or a subset of TAMs) in oncological contexts, their presence in noncancerous disorders, including neurodegenerative diseases, muscular dystrophies, and rheumatoid arthritis, among other diseases, indicates a broader role that is not limited to tumors. These macrophages appear to be more closely associated with chronic or prolonged inflammatory states rather than malignancy per se. This observation provides a rationale for reclassifying SPP1+ macrophages as a distinct subset characterized by their involvement in sustaining inflammation, remodeling extracellular matrices, and mediating immune responses. Such a redefinition has a greater potential to reflect the functional plasticity and cross‐disease relevance of these cells, thereby guiding research and therapeutic strategies targeting these cells.

## 2 CONCLUSION AND FUTURE DIRECTIONS

The emerging understanding of SPP1+ macrophages challenges the traditional view of these cells as mere bystanders in disease. Their consistent association with poor outcomes across diverse contexts positions them as critical regulators of pathology. Future studies should explore the mechanisms driving their differentiation and function, particularly in response to environmental cues such as hypoxia, lipid metabolism, and extracellular calcium. Furthermore, therapeutic interventions targeting SPP1 signaling pathways, either directly or through upstream regulators like CD44, could provide a means to mitigate their detrimental effects.

An exciting avenue for future research lies in unraveling the origins of this macrophage population. Notably, significant efforts have suggested their monocytic derivation,^11^ proposing that these cells are trapped in an intermediate activation state, simultaneously exhibiting both pro‐ and anti‐inflammatory signatures. This hybrid phenotype aligns with other findings and fits within the current understanding of macrophage polarization, which exists on a dynamic continuum rather than as fixed M1 (pro‐inflammatory) and M2 (anti‐inflammatory) extremes.^35^ A particularly compelling question is whether this hybrid state arises from incomplete differentiation or results from aberrant transcriptional programs shaped by disease‐specific environmental cues. Exploring these possibilities could deepen our understanding of macrophage biology and uncover novel therapeutic opportunities, making this an area of immense scientific potential.

In conclusion, SPP1+ macrophages represent a fascinating convergence of biology across aging, inflammation, and cancer. Their shared traits suggest they are not only indicators of disease severity but also potential nodes for therapeutic intervention, warranting further investigation into their biology and functional versatility.

## AUTHOR CONTRIBUTIONS

Conceptualization: Alessandro Palma; methodology: Alessandro Palma, Alessio Reggio; validation, data curation, visualization: Alessandro Palma, Alessio Reggio, Claudia Fuoco, Rebecca Deodati; writing – original draft, writing – review and editing: Alessandro Palma, Alessio Reggio, Claudia Fuoco, Rebecca Deodati; supervision and project administration: Alessandro Palma, Alessio Reggio.

## DISCLOSURES

The authors declare no conflict of interest.

## Data Availability

Data sharing is not applicable to this article as no datasets were generated or analyzed during the current study.
